# Do spouse burden of care, family resilience, and coping affect family function in gynecologic cancer in Korea?: a cross-sectional study

**DOI:** 10.4069/kjwhn.2022.08.03

**Published:** 2022-09-30

**Authors:** Minkyung Kim, Sukhee Ahn

**Affiliations:** College of Nursing, Chungnam National University, Daejeon, Korea

**Keywords:** Coping, Family functioning, Family resilience, Gynecologic cancer, Spouse

## Abstract

**Purpose:**

This study aimed to investigate family functioning among spouses of gynecologic cancer patients in Korea. McCubbin and McCubbin’s Family Resilience Model (1993) guided the study focus on burden of care, family resilience, coping, and family functioning.

**Methods:**

An online survey collected data from 123 spouses of gynecologic cancer patients through convenience sampling from online communities for gynecologic cancer patients in Korea. Burden of care, family resilience (social support, family hardiness, and family problem-solving communication), coping, and family functioning were measured by self-report.

**Results:**

The patients (44.7%) and their spouses (47.2%) were mostly in the 41 to 50-year age group. Stage 1 cancer was 44.7%, and cervical cancer was the most common (37.4%) followed by ovarian cancer (30.9%) and uterine cancer (27.6%) regarding the cancer characteristics of the wife. Family function, burden of care, family resilience, and coping were all at greater than midpoint levels. Family functioning was positively related with social support (r=.44, *p*<.001), family hardiness (r=.49, *p*<.001), problem-solving communication (r=.73, *p*<.001), and coping (r=.56, *p*<.001). Multiple regression identified significant factors for family functioning (F=25.58, *p*<.001), with an overall explanatory power of 61.7%. Problem-solving communication (β=.56, *p*<.001) had the greatest influence on family function of gynecologic cancer families, followed by coping (β=.24, *p*<.001) and total treatment period of the wife (β=.17, *p*=.006).

**Conclusion:**

Nurses need to assess levels of family communication and spousal coping to help improve gynecologic cancer patients’ family function, especially for patients in longer treatment.

## Introduction

Among gynecologic cancers in Korea, cervical cancer ranked first with 52.9% per 100,000 people, uterine cancer ranked second with 26.2%, and ovarian cancer ranked third with 20.9% [1]. As for the 5-year observed survival rate of gynecologic cancer, it has increased in all three cancers from the 1993–1995 period to the 2014–2018 period: from 75.7% to 78.1% for cervical cancer, 80.3% to 86.8% for uterine cancer, and 58.4% to 63.6% for ovarian cancer [1]. As the survival rate increases along with the increase in gynecologic cancer incidence, nurses need to help gynecologic cancer patients and their families effectively manage the long-term cancer treatment process [2].

Women with gynecologic cancer experience symptoms related to reproductive function, such as abdominal discomfort, urination and bowel dysfunction, vaginal dryness, and decreased sexual function [3]. In addition, gynecologic cancer patients experience difficulties in performing their role as mother and/or wife due to health problems [4]. As a result, gynecologic cancer patients may experience negative physical, psychological, and social reactions from the time of cancer diagnosis to the progression of the disease, treatment, and cure [3,4].

In gynecologic cancer patients, the family is not only a primary source of physical, mental, and social support but can also be an active participant in their treatment and recovery. In addition, considering the situation in Korea, where families often shoulder a large part of caring for patients, the family of gynecologic cancer patients can face various difficulties: such as imbalance of the family system due to the disease, role changes due to the patient’s condition, and lack of knowledge in patient care [5]. In addition, the experience of enduring pain from the treatment process and resulting patient complications have been reported to present burden of caring in family [6].

Family caregivers of cancer patients generally had to shoulder the responsibility to take care of their family despite their physical, mental, social, and spiritual suffering and economic difficulty [7]. Family members also experienced burden of caring due to psychological and economic instability [8], which became a source of stress [9]. If the burden of cancer patients’ family continues to accumulate, family tend to become passive in caring for the patient, which can lead to difficulties in family recovery, such as indifferent reactions to the patient’s pain, and can cause changes in family relationships or family function [10]. Higher resilience of patients and care providers is associated with better family functions and can exert a positive effect [11]. In addition, family problem-solving communication and coping can positively affect family functions [12], which can need to examine the family function according to burden of care in the family of gynecologic cancer patients.

McCubbin and McCubbin [13] defined family resilience as the ability of a family to adapt to stress and recover from adversity, and describe it as a family quality that allows families to adapt more positively in the face of a crisis. In their Family Resilience Model [14], social support, family strength, and problem-solving communication strategies are presented as factors of family resilience, and recovery and adaptation through interaction between these factors are emphasized. In particular, Korean culture is family-centered and has unique attributes based on collectivist culture, and considers relational values as very important. As such, the diagnosis and treatment process of gynecologic cancer greatly affects the entire family of cancer patients, and is a major crisis event in which the burden and stress felt as the main care provider increases [15]. Prior studies have reported a significant correlation between family resilience and family function of liver cancer patients and caregivers in China [12] and a direct and positive relationship between family function and resilience in Chinese lung cancer patients and caregivers [11]. While research on breast cancer patients and family is active in Korea [16,17], studies on gynecologic cancer patients are limited, even more so for family resilience and family function.

Families of gynecologic cancer patients use coping methods to maintain equilibrium against physical and mental difficulties while caring for the patient, and such coping methods differ from family member to family member [18]. While there are families who use active coping methods with the aid of social support systems to actively resolve the family’s crisis situation, other families may use emotional coping to reduce emotional pain in stressful situations [19]. Prior studies in Korea have focused on the relationship of stress, coping method, and burnout in family members caring for cancer patients [18], and the positive effect of breast cancer survivors’ family coping on family function [17]. However, there is a lack of studies on family coping and family function in gynecologic cancer patients and their family.

Of the studies conducted on gynecologic cancer patients in Korea, many focused on the patients’ health problems and quality of life [20-22]. While studies exploring family functions for gynecologic cancer patients have been confirmed overseas [23], little has been explored in Korea. In studies on families of general cancer patients in Korea [7-9], most of the family members were wives or adult children (female); and in the case of female cancers, two studies reported the main caregiver as the spouse. One evaluated the relationship between social support, family coping, and family function as perceived by spouses of breast cancer patients [17]. Another reported that when the spouse of a young breast cancer patient is actively involved in caring, difficulties such as lack of income, lack of social exchange, and lack of parental care in the child’s growth process can arise [16]. Although a study noted that spouses of Korean gynecologic cancer patients expressed struggles such as feeling sorry for the wife, regret for not having been more available, wanting to run away from having to watch the patient’s pain, and psychological stress from the loss of fertility [5], there is a sore lack of research on family functions. There were studies abroad that looked at differences in research variables according to general characteristics such as caregiver’s age, care-related characteristics such as nursing period [11], and studies that looked at differences between patients and spouses on characteristics of cancer patient’s disease in Korea [17]. Therefore, among the factors influencing the family function of cancer patients, research is needed to consider changes in occupational status, disease characteristics (cancer stage, total treatment period), and care characteristics (alternative manpower) of gynecologic cancer patients. Therefore, this study aimed to identify care burden, family resilience, coping, and family function in spouses of gynecologic cancer patients.

The purpose of this study was to understand the impact of burden of care, family resilience, and coping on family function in spouses of women with gynecologic cancer. Specific objectives were as follows:

1) To identify family function, spouse burden of care, family resilience, and coping

2) To identify family function according to spouses’ characteristics and patients’ disease-related characteristics

3) To determine the relationships among family function, burden of care, family resilience, and coping

4) To identify the factors that affect family function of spouses of gynecologic cancer patients

## Methods

Ethics statement: This study was approved by the Institutional Review Board of Chungnam National University (202107-SB-174-01). Informed consent was obtained from the participants.

### Research design

This study employed a correlational design using a cross-sectional survey, to identify the effects of burden of care, family resilience, and coping on family function in spouses of gynecologic cancer patients. This study adhered to the STROBE (Strengthening the Reporting of Observational Studies in Epidemiology) reporting guidelines (https://www.strobe-statement.org/).

### Sample

Spouses of women with gynecologic cancer who were undergoing surgery, chemotherapy, radiation therapy, and/or hormone therapy, or were under follow-up observation after treatment, were the target population. Inclusion criteria were spouses of aged 20 years or older who agreed to participate in the study. Cancer patients diagnosed with terminal cancer or under hospice treatment were excluded.

As a preceding study [17] reported that social support and family coping accounted for 42%, and a correlation r of .66, these were used to calculate the effect size for this study. Using the G*power program, significance level (α)=0.05, power (1–β)=0.8, and effect size f^2^=.15, and using 10 predictive factors (cancer stage, total treatment period, spouse occupational status change, number of children, presence or absence of alternative help if necessary, care burden, social support, family strength, problem-solving communication, coping), at least 118 participants were required. Considering a potential loss of 10% due to incomplete responses, 130 spouses were recruited via convenience sampling and 123 people (94.6%) who submitted complete data to the online survey were analyzed for the study.

### Measurement

All study instruments were used after contacting the developer of the original tool for their consent.

#### Burden of care

Burden of care was measured by the Caregiver Reaction Assessment Scale developed by Given et al. [24], using the Korean version [25]. This 24-item instrument consists of questions on self-esteem (seven questions), life pattern change (five questions), lack of family cooperation (five questions), economic burden (three questions), and physical burden (four questions). A 5-point Likert (1, absolutely not to 5, very much) is used and after reverse coding for some self-esteem items, scores are summed (possible range, 24–120). Higher scores indicate a greater degree of care burden. The internal reliability of the original tool [24] was .88 and in this study, it was .69.

#### Family resilience

Social support, family hardiness, and problem-solving communication were selected as constituting family resilience based on McCubbin and McCubbin’s [14] resilience model of family stress, coordination, and adaptation to disease stress-induced crises. This framework postulates that resilience is an internal and external resource of the family system, which has components including family strength, family management, family problem solving, and response strategies.

• Social support: The Korean version [26] of the 17-item Social Support Index (SSI) developed by McCubbin et al. [27] was used. Items are rated on a 5-point Likert (1, not at all to 5, very much so) and higher summed scores (possible range, 17–85) indicate a greater degree of social support. Cronbach’s α of the SSI [26] was .82, .75 in a study of Korean cancer patient families [27], and .73 in this study.

• Family hardiness: The Family Hardness Index (FHI) was developed by McCubbin et al. [28], and its Korean version [29] was used in this study. Each of the 20 questions is rated on a 4-point Likert (1, not at all to 4, very much so) and summed (possible range, 20–80). The higher the FHI score, the stronger the family. Cronbach’s α was .82 for the FPSC [28], .91 in a Korean study [29], and .83 in this study.

• Problem-solving communication: The Korean version [27] of the Family Problem Solving Communication (FPSC) [30] was used. Two types of communication are identified: ‘aggressive problem-solving communication’ that tends to worsen stress situations or ‘positive problem-solving communication’ that conveys support and interest and aims for a calming effect. The 10 questions are rated on a 4-point scale (1, not at all to 4, very much) and summed (possible range, 10–40). Cronbach’s α of the original tool [30] was .89, .76 in a Korean study [27], and .75 in this study.

#### Coping

The 69-item Ways of Copying (WOC) developed by Lazarus and Folkman [19] was translated into Korean [31] and its modified 24-item version [32] was used. This WOC has 12 questions on active coping (six for problem-oriented coping, six for social support pursuing coping) and 12 questions on passive coping (six for emotion-oriented coping, six for aesthetic thinking coping). Rated on a 4-point Likert (1, not used to 4, used very much), scores are summed (possible range, 24–96) and higher scores mean that particular coping style is used more often. Cronbach’s α for active coping and passive coping were .86 and .76, respectively, for the original tool [18], .80 and .69 in a previous study [32], and .84 and .75. in this study. The Cronbach’s α for active and passive coping altogether was .87 in this study.

#### Family function

The Korean Family Functioning Scale [33], which measures family function under the stress of chronic disease in family members, was used. This 26-item tool consists of six subareas: affective cohesion of family functions, relationship with external resources, family norms, roles and responsibilities, communication, and financial resources. A 4-point Likert (1, not at all to 4, very much so) is used for summed scores (possible range, 26–104). Higher scores indicate better family function. Cronbach’s α of the original tool [33] was .87 and .90 in this study.

### Characteristics of spouses and patients

Based on the literature, the following were assessed: spouse’s age, education level, occupational status change, monthly income, and number of children. For disease-related characteristics of the gynecologic cancer patient, spouse was asked about wife’s age, cancer type, cancer stage, recurrence, total treatment period, and treatment type were investigated. Also, the following care characteristics were assessed: existence of alternative help, alternative help providers, period and time of caring for the wife, changes in spouse’s life after diagnosis of the cancer, presence of spouse’s health problems, person covering medical costs, and monthly medical expenses.

### Data collection

Data collection for this study was conducted from October 20, 2021 to January 30, 2022 from three online communities for gynecologic cancer patients in Korea. After obtaining permission from the administrator of the online community, a recruitment flyer was posted and interested participants could voluntarily contact the research team. After screening for eligibility, potential participants were sent a link to the online questionnaire. The survey took 15 to 20 minutes and to those who chose to share personal contact information, a mobile gift card (worth approximately 4 US dollars) was provided after completing the questionnaire.

### Data analysis

Using IBM SPSS Statistics for Windows, ver. 26.0 (IBM Corp., Armonk, NY, USA) with the significance level set at *p*<.05, descriptive statistics were done for the spouse’s general characteristics, the patient’s disease-related characteristics, care characteristics, burden of care, family resilience, coping, and level of family function. Differences in family function according to the characteristics of spouse and patient were analyzed by independent t-test and one-way analysis of variance, and the Scheffé test was used for post-analysis. Pearson correlation coefficient was calculated for the relationships among care burden, family resilience, coping, and family function. Finally, hierarchical multiple regression analysis was performed to identify the factors influencing family function.

## Results

### Characteristics of the sample

#### Characteristics of gynecologic cancer patients

Most patients were in the 41 to 50 years of age category (n=55, 44.7%). Cervical cancer was the most common (n=46, 37.4%), followed by ovarian cancer (n=38, 30.9%) and uterine cancer (n=34, 27.6%). Stage I gynecologic cancer was close to half (n=55, 44.7%) and 90.2% (n=111) were not recurred cancer. Treatment duration was mainly 6-12 months (n=42, 34.1%) or 3-6 months (n=38, 30.9%), followed by >13 months (n=27, 22.0%). For treatment, most had experienced surgery (n=88, 71.7%), as well as chemotherapy (n=62, 50.8%) and radiation (n=37, 30.8%); some had received target therapy (n=19, 15.8%), and antihormonal therapy (n=15, 11.7%) (Table 1).

#### Spouse characteristics

Similar to the patient, most spouses were in the 41 to 50-year age range (n=58, 47.2%). Most were college graduates (n=102, 82.9%) and 1/4 had experienced work changes since their wife’s diagnosis of cancer (n=30, 24.4%), e.g., being absent from work, leaving early, or resigning. Monthly household income was in the 3 million to 5 million Korean Won (KRW) category (approximately 2,400–4,000 US dollars) for roughly half (n=62, 50.4%), which is comparable to the national household average for 2021 (4.73 million KRW) [34] and one child was the most common (n=75, 61.0%) (Table 1).

#### Care-related characteristics

Roughly 2/3 (n=82, 66.7%) were able to seek alternative help when caregiving support was needed, of which 57.3% (n=47) were their parents. The number of months of caring for the wife was 1 to 6 months (n=47, 39.2%), and 1 to 5 hours was most common (n=88, 74.6%). Among the changes in spouse’s life after the diagnosis of gynecologic cancer, lack of leisurely time was most common (n=39, 40.2%), followed by having new appreciation for wife and family (n=26, 26.8%). Physical/mental burdens and housework/childcare burdens (both n=13, 13.4%), as well as economic burdens (n=6, 6.2%) were also noted. Seventeen spouses (13.8%) had health problems and medical costs were mostly covered by the spouse (n=113, 91.7%) although parents or siblings also participated (n=14, 11.7%). About half (n=68, 55.3%) responded their monthly medical expenses were in the 1 million to 3 million KRW range (roughly 800–2,400 US dollars), and 17 people (13.8%) in the 3 million won to 5 million KRW (roughly 2,400–4,000 US dollars) range (Table 1).

### Family function, family burden of care, family resilience, and coping

Family function was 75.83±9.34 points out of 104 points, which was higher than midpoint. The burden of care perceived by the spouse was 74.00±7.72 points out of 120 points, which was higher than midpoint. For family resilience, (1) social support was 57.85±6.89 points out of 85 points, suggesting moderate or higher level; (2) family hardiness was 58.45±6.75 points out of 80, suggesting moderate or higher level; and (3) problem-solving communication was 26.05±2.07 points out of 40, suggesting moderate or higher level. Coping was 64.54±9.37 points out of 96 points suggesting moderate or higher level (Table 2).

### Differences in main variables according to the characteristics of spouse and patient

The burden of care (F=2.49, *p*=.047) and coping (F=2.49, *p*=.047) differed according to the spouse’s age. Spouses who had experienced work changes had significantly higher care burden (t=3.22, *p*=.002), used more coping (t=2.25, *p*=.026), and had higher family function scores (t=2.72, *p*=.007) compared to spouses with no changes.

For the subcomponents of family resilience, there was a significant difference in social support by monthly income, and in family strength by number of children. Spouses with monthly income of 3 million to 5 million KRW (roughly 2,400-–4,000 US dollars) had better social support than those in the <3 million KRW (< 2,400 US dollars) range by post-hoc test (F=3.44, *p*=.035). Also by post-hoc analysis, families with two children showed higher family hardiness than those with one child (F=5.46, *p*=.005).

Among the wife’’s disease characteristics, the burden of care differed by gynecologic cancer stage (F=3.01, *p*=033). Family resilience in social support, family hardiness, and family function also differed by duration of the total treatment period. Post-hoc test of the total treatment period found that the degree of social support was higher in cases of >1 year than those of 3- to 6 months (F=4.48, *p*=.005); the degree of family hardiness was higher in cases of ≥13 months compared to those of 7- to 12 months (F=3.54, *p*=.017); and family function was higher in cases of ≥13 months compared to 7 to -12 months (F=4.14, *p*=.008). Also, burden of care, problem-solving communication, and coping were significantly different by monthly medical expenses (F=3.29, *p*=.023). Post-hoc test of monthly medical expenses show that burden of care was higher in cases of 1 million to 3 million KRW (roughly 800-–2,400 US dollars) (F=4.58, *p*=.005); and women in 1 million to 3 million KRW used more problem-solving communication than those in the < 1 million KRW (<800 US dollars) group (F=3.82, *p*=.012).

As for care characteristics, there were significant differences in social support, family strength, problem-solving communication, and family function according to availability of alternative help when needed. Those who had alternative help had higher social support (t=2.64, *p*=.009), greater family hardiness (t=2.62, *p*=.010), problem-solving communication (t=3.09, *p*=.002), and family function (t=2.02 and, *p*=.048) compared to their counterparts. The number of months of caring for wife was also significantly related to burden of care, social support, family strength, and coping. Spouses who cared for their wife for 7- to 12 months had a greater burden of care than those with 1~ to months of caring (F=5.93, *p*=.004). Spouses with ≥13 months of caring had more social support than those with 1- to 6 months (F=3.51, *p*=.033), higher family hardiness than those with 7- to 12 months (F=4.67, *p*=.011), and a higher degree of family coping than those with 1- to 6 months of caring (F=3.21, *p*=.044) (Table 3).

### Relationships among family care burden, family resilience, coping, and family function

Family function in gynecologic cancer patient’s family was positively correlated with social support (r=.44, *p*<.001), family hardiness (r=.49, *p*<.001), problem-solving communication (r=.73, *p*<.001), and coping (r=.56, *p*<.001). However, burden of care was not significantly related to family resilience, coping, or family function. Family coping had a positive weak correlation with social support (r=.33, *p*<.001), family hardiness (r=.27, *p*=.002), and problem-solving communication (r=.43, *p*<.001). Among the subcomponents of family resilience, social support had a positive moderate correlation with family strength (r=.68, *p*<.001), problem-solving communication (r=.51, *p*<.001), and family hardiness (r=.62, *p*<.001) (Table 4).

### Factors affecting family function

Before multiple regression analysis, suitability of the data was confirmed through the assumption of the regression equation (normality, linearity multicollinearity) and residual diagnosis (normality of residuals, independence of errors, and equal variance). The Durbin-Watson value was 1.85, which was close to reference 2, securing the independence of the error. The tolerance limit (0.42–0.97) and the variance inflation factor (VIF), which was in the range of 1.02 to 2.37, indicated no problem in multicollinearity of the independent variables.

In Model 1, characteristics that were significant for family function were entered, i.e., spouse’s work change, total treatment period of wife, and alternative help when needed. This model was significant (F=7.14, *p*<.001), with an explanatory power of 13.1%. Of the variables, spouse’s work change (β=.18, *p*=.036) and total duration of treatment (β=.26, *p*=.004) affected family function. Adding burden of care in Model 2, the total treatment period of the wife (β=.19, *p*=.003) and alternative help when needed (β=.18, *p*=.044) predicted family function with an explanatory power of 14.1% (F=6.03, *p*<.001). Further adding social support, family hardiness, and problem-solving communication, as subcomponents of family resilience, in Model 3, the total duration of treatment for the wife (β=.19, *p*=.003) and problem-solving communication (β=.65, *p*<.001) had an explanatory power of 57.7% for family function (F=24.55, *p*<.001). In Model 4, coping was added and the final regression model found that problem-solving communication (β=.56, *p*<.001) had the greatest influence on family function of gynecologic cancer families, followed by coping (β=.24, *p*<.001) and total treatment period of the wife (β=.17, *p*=.006). In other words, the higher the problem-solving communication, the higher coping, and total treatment period of ≥1 year, the higher family function of the gynecologic cancer patient’s family could be expected. Model 4 was significant (F=25.58, *p*<.001) with an explanatory power of 61.7% (Table 5).

## Discussion

The main factors affecting family function of gynecologic cancer patients identified through hierarchical multiple regression analysis were problem-solving communication, coping, and total treatment period of ≥1 ≥1 year. Social support, family hardiness, and problem-solving communication, which were subareas of family resilience, were related to family function; and problem-solving communication was found to be the most important influencing factor on family function. These findings are in line with a previous study on families of cancer patients, that found level of social support of care providers had a significant positive correlation with their resilience [35], and another study on breast cancer survivors [29], which reported that higher family resilience was related to more use of problem-solving communication patterns. Our study provides further evidence for the literature on family function being linked to high resilience in families of terminal cancer patients [35], and family hardiness directly affecting family function in families of children with cancer [27]. As family communication is an important part of cognitive family function [33], our findings emphasize the importance of open communication within the family dealing with gynecologic cancer. As such, nursing interventions to strengthen family resilience can help affirm internal resources and improve family function through external support, such as a family resilience program for gynecologic cancer patients and family.

The level of coping of spouses was the second most significant influential factor on family function in gynecologic cancer. In the correlation analysis, coping had a positive relationship with social support, family hardiness, and problem-solving communication, which are subfactors of family resilience. These findings further support prior literature that family hardiness supported caregiver’s positive coping in families of breast cancer patients [36] and that more social support indirectly affected family coping in families of breast cancer patients [17]. Our study also adds to existing knowledge from a prior study on breast cancer survivors, which found that the more positive patterns of problem-solving communication were used by the family, the more likely problem-solving and behavioral-coping strategies were used [29]. Therefore, nurses should be able to provide family education and counseling approaches that can improve the family’s coping skills in the midst of gynecologic cancer.

This study is also consistent with reports that the longer the treatment period of gynecologic cancer, the more positive effect on family function improvement [17], and that having alternative help available was linked with higher social support and lower care burden in families of cancer patients [37]. Given that the uncertain nature and progress of gynecologic cancer often requires long-term treatment and may cause changes in family function, this further underscores the need for supportive care of the family [38] and more studies that closely examine family function in gynecologic cancer.

On the other hand, there was no independent effect of the burden of care on gynecologic cancer family’s function, nor were there significant correlations with other independent variables. This finding differs from a previous study that reported a significant negative correlation of moderate strength between family function and family burden [39]. Our result may be interpreted that while spouses indeed have burden of care when the wife has gynecologic cancer, it does not appear to affect changes in family function, which may be related to family dynamics.

The burden of care in our sample was an average of 74.00±7.72 points out of 120 points, showing a higher level of burden compared to the ‘normal’ level of care burden of 72 points [25]. Considering the age profile of the spouses (most in the 41–50-year age group), that 24.4% had experienced work changes, 68% had monthly medical expenses of <3 million KRW, and 39.2% had a care period for 1 to 6 months, these factors to have increased the burden of caring. This level of care burden is similar to a study on spouses of breast cancer patients [16], which reported a moderate burden of care. As such, it seems that spouses of female cancer patients have a burden of care not only due to the wife’s cancer treatment but also linked to having to take on child rearing and housework-related care activities. However, since the burden of care may vary depending on family resilience [16,27,28,40-42], coping [18,38,43], and other characteristics of care [16,17,29,40,41,44,45], further study is necessary to explore these factors.

The levels of family resilience in our samples were all at moderate or higher scores for social support, family hardiness, and problem-solving communication. This is similar to another study that reported greater than midpoint scores of social supports of families of cancer patients that included breast cancer, uterine cancer, and ovarian cancer [40]. It also aligns with another study that found breast cancer patients’ spouses solve problems through social support asking for help to relieve the burden of multiple roles [41], and another study on spouses of young women with breast cancer [16] in which family support was high among social support subdomains. This suggests that when faced with stress and family crisis stemming from cancer, the family of gynecologic cancer patients has the resources to ask for help from neighboring groups and social. The level of family hardiness in this study is also similar to the moderate or higher level reported for cancer patient families [42]. This is because the strength of the family is the family’s resistance to stress and an adaptive resource, and supports how internal strength and durability of the family can overcome life tensions [28]. Finally, the level of problem-solving communication in this study was also similar to that in pediatric cancer families [27]. Spouses of gynecologic cancer patients appear to overcome the family’s difficulties by promoting family solidarity through communication, particularly aimed at problem solving.

For gynecologic cancer families, coping level was also greater than midpoint, similar to the level of coping reported for families of patients with various cancers, such as digestive, respiratory and genitourinary cancers [18]. Family function was also above medium level and was similar to the family function level reported by Korean spouses of breast cancer patients [43].

In this study, spouses noted significant differences in work status changes in caring burden, coping, and family function. Being absent from work or leaving work early to care for family may result in economic loss and possible medical cost burden [16,40]. Accordingly, if the spouse is the main care provider, the longer period of caring for the wife and greater burden of care such as housework, can lead to work changes. Our finding that the total treatment period affected significant differences in social support, family hardiness, and family function, supports a previous study that a longer treatment period makes the need for a social support system and stronger ties within the family all the more important, which is needed to strengthen overall family function [17]. As for the significant differences in burden of care, social support, family hardiness, and coping according to the number of months of caring for wife, considering that family caregivers of cancer patients are required to learn adaptive care behaviors along the cancer process and face many difficulties without any preparation [44], as the wife’s care is prolonged, the spouse tries to relieve the burden of multiple roles by seeking social support [41]. Family hardiness also acts as the internal strength and durability in cancer patient families, especially with increasing care periods, and can be a driving force to overcome the crisis by cooperating with each other [29]. Our finding on gynecologic cancer spouses is similar to prior research on breast cancer, which found significant differences in spouse burden and coping according to the treatment stage, with spouses using more coping behaviors to reduce caring burden as caregiving months increased [45].

Among the characteristics related to care, there were significant differences in social support, family hardiness, problem-solving communication, and family function according to availability of alternative help when needed. This is similar to the result that family hardiness and communication improved when there were three caregivers as opposed to one [37]. Given that in families with high family function, more family members participate in the care of patients [37], alternative help from within and outside the family can be a great source of social support.

Thus, assessing whether supportive help is available may be needed to understand and promote family function, in addition to the possibility of change in spouse’s work patterns, and disease and treatment characteristics of gynecologic cancer patients.

This study focused specifically on spouses of gynecologic cancer patients rather than vaguely observing ‘family’ and was thus able to suggest directions for a spouse-focused family function study. However, as this study was conducted through convenience sampling through internet communities related to cancer, representation of the target sample may be insufficient. Also burden of care was measured as limited to the spouse’s experience, this may have affected why burden of care was not an influential factor for family function of gynecologic cancer patients. As such, future research that includes other family members’ role and caregiving burden is needed. Since the degree of care burden may vary depending on the cancer stage and treatment mode, future studies may also benefit from recruiting participants according to treatment completed and/or ongoing treatment status to better understand the burden of care and family function.

In conclusion, in order to help family function of gynecologic cancer patients, it is necessary to first assess the spouse’s care burden, and in particular, to check level of family resilience, coping, and family function; as an integrative way to see whether the couple with gynecologic cancer and family members function as a single system. In addition, strengthening the family’s individual support system by checking the degree of mobilizing support from relatives, neighbors, friends, colleagues, and public social services can reduce the burden on cancer families. This study confirmed that spouses of gynecologic cancer patients can have a positive effect on family function through positive communication and coping focused on problem-solving. Therefore, nurses can use findings to develop appropriate programs aimed at improving problem-solving communication and coping skills between the gynecologic cancer patient and spouse during the long-term treatment process.

## Figures and Tables

**Table 1.** Characteristics of gynecologic cancer patients and spouses (N=123)

Variable	Categories	n (%)
*Characteristics of wives*		
Age (year)	20–30	3 (2.5)
	31–40	49 (39.8)
	41–50	55 (44.7)
	51–60	11 (8.9)
	>60	5 (4.1)
Type of cancer	Cervical cancer	46 (37.4)
	Ovarian cancer	38 (30.9)
	Uterine cancer	34 (27.6)
	Others^‡^	5 (4.1)
Cancer stage	1	55 (44.7)
	2	42 (34.1)
	3	17 (13.8)
	4	9 (7.3)
Recurrence of cancer	No	111 (90.2)
	Yes	12 (9.8)
Total treatment period (month)	<3	16 (13.0)
	3–6	38 (30.9)
	6–12	42 (34.1)
	>12	27 (22.0)
Treatment modality^†^	Surgery	88 (71.7)
	Chemotherapy	62 (50.8)
	Radiation therapy	37 (30.8)
	Targeted therapy	19 (15.8)
	Hormone therapy	15 (11.7)
*Characteristics of spouses*		
Age (year)	20–30	3 (2.4)
	31–40	40 (32.5)
	41–50	58 (47.2)
	51–60	15 (12.2)
	>60	7 (5.7)
Level of education	<High school	21 (17.1)
	≥College	102 (82.9)
Work status change	No	93 (75.6)
	Yes	30 (24.4)
Monthly family income (KRW)	<3 million	28 (22.8)
	3-5 million	62 (50.4)
	>5 million	33 (26.8)
Number of children	1	75 (61.0)
	2	43 (35.0)
	3	5 (4.1)
*Characteristics of care*		
Alternative help when needed	No	41 (33.3)
	Yes	82 (66.7)
	Parents	47 (57.3)
	Children	26 (31.7)
	Brothers, sisters	5 (6.1)
Duration of caring for wife (months) (n=120)	1–6	47 (39.2)
	7–12	45 (36.6)
	>13	28 (22.8)
Time spent caring for wife (hours/day) (n=118)	1–5	88 (74.6)
	≥6	30 (25.4)
Changes in spouse’s life after cancer diagnosis (n=97)	Lack of free time	39 (40.2)
	Change in life values	26 (26.8)
	Physical and mental burden	13 (13.4)
	Housework and childcare burden	13 (13.4)
	Economic burden	6 (6.2)
Own health problems	No	106 (86.2)
	Yes	17 (13.8)
Family paying medical bills^†^	Spouse	113 (91.7)
	Patient	23 (19.2)
	Parents, brothers, sisters	14 (11.7)
	Children	7 (5.8)
Monthly medical expenses (KRW)	<1 million	35 (28.5)
	1–2.9 million	68 (55.3)
	3–4.9 million	17 (13.8)
	≥ 5 million	3 (2.4)

KRW: Korean won (1 million is approximately 800 US dollars).

† Multiple response;

‡ fallopian tube cancer, vaginal cancer.

**Table 2.** Level of family functioning, burden of care, family resilience, and coping (N=123)

Variable	Mean±SD	Possible range	Min	Max
Family functioning	75.83±9.34	26–104	51.00	99.00
Burden of care	74.00±7.72	24–120	48.00	94.00
Family resilience				
Social support	57.85±6.89	17–85	45.00	80.00
Family hardiness	58.45±6.75	20–80	44.00	76.00
Problem-solving communication	26.05±2.07	10–40	20.00	31.00
Coping	64.54±9.37	24–96	31.00	88.00

**Table 3.** Comparison of family functioning, burden of care, family resilience and coping according to characteristics (N=123)

Variable	Categories	Family functioning		Burden of care		Family resilience						Coping	
						Social support		Family hardiness		Problem-solving communication			
		Mean±SD	t/F (*p*)	Mean±SD	t/F (*p*)	Mean±SD	t/F (*p*)	Mean±SD	t/F (*p*)	Mean±SD	t/F(*p*)	Mean±SD	t/F (*p*)
*Characteristics of spouses*													
Age (year)	20–30^a^	72.33±11.55	0.43	71.67±8.62	2.49	55.67±6.66	0.21	61.00±1.00	1.74	26.33±5.51	0.69	53.67±3.06	2.49
	31–40^b^	74.58±11.10	(.789)	75.97±6.21	(.047)	57.60±6.24	(.932)	56.48±6.23	(.147)	28.93±3.65	(.602)	62.88±8.93	(.047)
	41–50^c^	76.57±8.65		74.12±7.71		58.36±7.74		59.91±6.90		29.29±3.53		66.38±9.20	
	51–60^d^	77.00±8.25		71.93±7.79		57.13±5.54		57.80±6.76		28.93±3.47		62.33±11.18	
	>60^e^	75.86±5.93		67.14±11.67		57.43±6.97		57.86±7.90		30.29±4.23		68.29±5.41	
Work status change	Yes	79.77±8.86	2.72	77.80±6.94	3.22	56.47±6.67	–1.27	58.53±7.24	0.08	30.07±3.39	1.67	67.83±8.85	2.25
	No	74.56±9.18	(.007)	72.77±7.60	(.002)	58.29±6.93	(.208)	58.42±6.62	(.936)	28.81±3.67	(.098)	63.48±9.33	(.026)
Monthly income (KRW)	<3 million^a^	75.00±5.84	0.21	72.25±7.46	0.47	55.04±5.67	3.44	57.39±4.75	0.57	28.86±3.25	0.11	62.39±8.33	1.29
	3–5 million^b^	75.81±10.10	(.808)	74.35±7.20	(.625)	59.06±7.28	(.035)	59.02±7.21	(.567)	29.13±3.66	(.893)	64.61±9.61	(.279)
	>5 million^c^	76.58±10.40		74.39±8.93		57.94±6.54	b>a^†^	58.27±7.34		29.30±3.97		66.24±9.63	
Number of children	1^a^	74.76±9.89	1.77	75.04±6.56	1.78	57.11±6.21	1.99	56.91±6.09	5.46	28.60±3.59	2.14	63.79±10.16	1.53
	2^b^	77.05±8.05	(.175)	72.30±9.52	(.172)	58.56±7.69	(.141)	60.72±7.34	(.005)	30.02±3.62	(.122)	65.12±7.74	(.222)
	3^c^	81.40±9.76		73.00±4.30		62.80±8.01		62.00±4.64	b>a^†^	29.00±3.54		71.00±8.46	
*Characteristics of wives*													
Cancer stage	1^a^	75.24±8.33	1.62	72.73±6.80	3.01	58.20±7.01	0.45	58.24±6.49	1.70	28.85±3.45	0.72	62.56±7.80	2.02
	2^b^	74.45±9.74	(.189)	76.02±7.37	(.033)	56.93±5.67	(.718)	57.31±5.85	(.171)	28.88±3.38	(.544)	66.57±8.98	(.115)
	3^c^	79.59±8.90		75.65±10.09		59.00±8.39		59.88±8.92		30.12±4.33		66.94±12.09	
	4^d^	78.78±12.89		69.22±7.16		57.78±8.81		62.33±6.87		29.89±4.60		62.67±12.56	
Total treatment period (month)	<3^a^	74.19±10.64	4.14	72.88±6.93		60.25±7.66	4.48	59.88±6.94	3.54	28.69±4.63	0.77	61.63±8.33	1.69
	3–6^b^	74.97±6.93	(.008)	73.00±8.55	1.24	55.26±5.32	(.005)	57.16±6.10	(.017)	28.97±3.52	(.513)	63.76±8.44	(.173)
	7–12^c^	73.81±9.62	d>c^†^	75.86±6.34	(.297)	57.38±7.05	d>b^†^	57.02±7.23	d>c^†^	28.81±3.75		64.31±9.74	
	>12^d^	81.15±9.54		73.19±8.73		60.78±6.89		61.63±5.73		30.04±2.90		67.74±10.20	
Monthly medical expenses (KRW)	<1 million^a^	72.57±10.80	2.10	70.20±9.34	4.58	57.71±6.87	0.24	57.06±7.28	0.94	27.49±4.06	3.82	62.74±8.29	3.29
	1–2.9 million^b^	77.34±8.70	(.104)	75.43±5.87	(.005)	58.24±7.38	(.866)	59.26±6.66	(.425)	29.96±3.25	(.012)	66.49±8.75	(.023)
	3–4.9 million^c^	76.29±7.22		75.18±7.86	b>a^†^	56.71±5.44		57.76±5.87		29.06±2.90	b>a^†^	62.47±9.77	
	≥5 million^d^	77.00±10.82		79.33±11.50		57.00±3.46		60.00±7.00		29.33±5.51		53.33±21.13	
*Care characteristics*													
Alternative help when needed	Yes	77.18±7.64	2.02	73.21±7.04	–1.62	58.98±6.76	2.64	59.55±6.40	2.62	29.80±3.34	3.09	64.85±10.13	0.52
	No	73.12±11.69	(.048)	75.59±8.81	(.108)	55.59±6.66	(.009)	56.24±6.96	(.010)	27.73±3.83	(.002)	63.93±7.70	(.607)
Duration of caring for wife (month) (n=120)	1–6^a^	74.11±6.63	2.57	72.64±8.18	5.93	56.53±6.38	3.51	57.87±6.36	4.67	28.66±3.61	1.20	62.47±7.41	3.21
	7–12^b^	75.82±10.61	(.081)	77.20±6.23	(.004)	57.47±6.61	(.033)	56.91±6.97	(.011)	29.18±3.60	(.305)	65.13±9.21	(.044)
	>12^c^	79.07±10.30		72.21±7.27	b>a^†^	60.71±7.48	c>a^†^	61.64±6.37	c>b^†^	30.00±3.69		67.89±11.15	c>a^†^

KRW: Korean won (1 million is approximately 800 US dollars).

† Scheffé test.

**Table 4.** Relationships among study variables (N=123)

Variable		r (*p*)					
		Burden of care	Family resilience			Coping	Family functioning
			Social support	Family hardiness	Problem -solving communication		
Family resilience	Social support	.01 (.964)	1				
	Family hardiness	–.11 (.225)	.68 (<.001)	1			
	Problem-solving communication	.01 (.918)	.51 (<.001)	.62 (<.001)	1		
Coping		.17 (.063)	.33 (<.001)	.27 (.002)	.43 (<.001)	1	
Family functioning		.14 (.130)	.44 (<.001)	.49 (<.001)	.73 (<.001)	.56 (<.001)	1

**Table 5.** Factors influencing family functioning (N=123)

Factor		Model 1		Model 2		Model 3		Model 4	
		β	t (*p)*	β	t (*p)*	β	t (*p)*	β	t (*p)*
Job status change^†^		.18	2.12 (.036)	.14	1.54 (.126)	.09	1.32 (.188)	.06	.87 (.384)
Total treatment period^†^		.26	2.98 (.004)	.27	3.12 (.002)	.19	3.06 (.003)	.17	2.78 (.006)
Alternative help when needed^†^		.15	1.78 (.078)	.18	2.04 (.044)	–.00	–0.07 (.945)	.02	.31 (.760)
Burden of care				.14	1.56 (.122)	.12	1.83 (.070)	.09	1.49 (.140)
Family resilience	Social support					.06	.74 (.460)	.01	.16 (.877)
	Family hardiness					.01	.12 (.902)	.03	.38 (.704)
	Problem-solving communication					.65	8.23 (<.001)	.56	7.11 (<.001)
Coping								.24	3.71 (<.001)
F (*p*)		7.14 (<.001)		6.03 (<.001)		24.55 (<.001)		25.58 (<.001)	
Adjusted R^2^		.131		.141		.575		.617	
ΔAdjusted R^2^ (*p*)				.010 (.122)		.434 (<.001)		.042 (<.001)	

† The indicator groups were as follows: job status change (yes), total treatment period (> 1 year), and alternative help when needed (yes).
